# Supplementary material for: Analysis of PPARγ Signaling Activity in Psoriasis
Source: Int J Mol Sci. 2021 Aug 10;22(16):8603. doi: 10.3390/ijms22168603 (PMC8395241; doi:10.3390/ijms22168603)
Supplement: Supplementary file 1 [file ijms-22-08603-s001.zip › Supplemental materials_Analysis of PPARg signaling activity in psoriasis/Pathway models/Models images and html files/Interleukin-17 and interleukin-22 signaling in psoriasis/6821.html]

KRT10


# Protein KRT10

|  |  |
| --- | --- |
| URN | urn:agi-llid:3858 |
| Total Entities | 0 |
| Connectivity | 737 |
| Name | KRT10 |
| Description | keratin 10 |
| Notes | This gene encodes a member of the type I (acidic) cytokeratin family, which belongs to the superfamily of intermediate filament (IF) proteins. Keratins are heteropolymeric structural proteins which form the intermediate filament. These filaments, along with actin microfilaments and microtubules, compose the cytoskeleton of epithelial cells. Mutations in this gene are associated with epidermolytic hyperkeratosis. This gene is located within a cluster of keratin family members on chromosome 17q21. [provided by RefSeq, Jul 2008] |
| Primary Cell Localization | Cytoplasm |

---

|  |  |
| --- | --- |
| Pathway | Proteins Involved in Ichthyosis |
|  | Ichthyosis Vulgaris |
|  | Genes with Mutations Associated with Ichthyosis |
|  | IL22 Induces Keratinocyte Proliferation in Psoriasis |
|  | Proteins with Altered Expression in Psoriasis |
|  | EGFR -> Expression Targets in Skin |
|  | Elevated Receptors -> Expression Targets in Skin |
|  | NOTCH -> Expression Targets in Skin |
|  | 2\_Interleukin-17 and interleukin-22 signaling in psoriasis |
|  | 1\_Ichthyosis vulgaris overview |
|  | Interleukin-17 and interleukin-22 signaling in psoriasis |

---

|  |  |
| --- | --- |
| MedScan ID | 3858 |

---

|  |  |
| --- | --- |
| LocusLink ID | 3858 |

---

|  |  |
| --- | --- |
| Alias | Keratin-10 |
|  | D130054E02 |
|  | KA10 |
|  | keratin 10, type I |
|  | Keratin X |
|  | epidermal keratin 10 |
|  | OTTMUSP00000005149 |
|  | Keratin, type I cytoskeletal 10 |
|  | KRT10 |
|  | keratin 10 (epidermolytic hyperkeratosis, keratosis palmaris et plantaris) |
|  | K1C I |
|  | type I keratin KA10 |
|  | cytokeratin 10 |
|  | CG15306-PA |
|  | 56 kDa cytokeratin |
|  | suprabasal cytokeratin 10 |
|  | CK 10 |
|  | RP23-336D20.2 |
|  | Krt1 X |
|  | Krt1 Xs |
|  | keratin complex 1, acidic, gene 10 |
|  | K10 |
|  | epidermal keratin X |
|  | BIE |
|  | EHK |
|  | KPP |
|  | BCIE |
|  | CK10 |
|  | CK-10 |

---

|  |  |
| --- | --- |
| GO ID | 0046982 |
|  | 0030280 |
|  | 0070268 |
|  | 0031424 |
|  | 0030216 |
|  | 0018149 |
|  | 0045684 |
|  | 0051290 |
|  | 0009986 |
|  | 0001533 |
|  | 0005737 |
|  | 0005829 |
|  | 0070062 |
|  | 0005615 |
|  | 0005882 |
|  | 0016020 |
|  | 0005634 |
|  | 0071277 |
|  | 0045095 |

---

|  |  |
| --- | --- |
| KEGG ID | hsa:3858 |

---

|  |  |
| --- | --- |
| Organism | Homo sapiens {Organism urn:agi-taxid:9606} |
|  | Homo sapiens |

---

|  |  |
| --- | --- |
| OMIM ID | 148080 |
|  | 113800 |
|  | 609165 |
|  | 607602 |

---

|  |  |
| --- | --- |
| Hugo ID | 6413 |
|  | HGNC:6413 |

---

|  |  |
| --- | --- |
| Human chromosome position | 17q21.2 |
|  | 17q21 |

---

|  |  |
| --- | --- |
| Swiss-Prot Accession | P13645 |
|  | P13645.6 |
|  | Q14664 |
|  | Q8N175 |

---

|  |  |
| --- | --- |
| PIR ID | A31994 |
|  | S02158 |

---

|  |  |
| --- | --- |
| GenBank ID | NC\_000017 |
|  | XM\_005257343 |
|  | XP\_005257400 |
|  | NM\_000421 |
|  | NP\_000412 |
|  | NG\_008405 |
|  | AC090283 |
|  | CH471152 |
|  | EAW60680 |
|  | EAW60681 |
|  | L20218 |
|  | AAB59438 |
|  | L20219 |
|  | AAB59439 |
|  | X14487 |
|  | CAA32649 |
|  | BC034697 |
|  | AAH34697 |
|  | CU455823 |
|  | J04029 |
|  | AAA60544 |
|  | M19156 |
|  | AAA59468 |
|  | M77663 |
|  | AAA59199 |
|  | P13645 |
|  | NG\_033147 |
|  | NC\_018928 |
|  | AMYH02033404 |
|  | DQ893356 |
|  | ABM84282 |
|  | DQ896675 |
|  | ABM87674 |
|  | AC\_000149 |
|  | ABBA01031821 |

---

|  |  |
| --- | --- |
| Swiss-Prot ID | K1C10\_HUMAN |

---

|  |  |
| --- | --- |
| Cell Localization | Secreted |
|  | Extracellular space |
|  | Cell surface |

---

|  |  |
| --- | --- |
| Ensembl ID | ENSG00000186395 |
|  | ENSP00000269576.5 |
|  | ENST00000269576.6 |
|  | ENSP00000269576 |
|  | ENST00000269576 |

---

|  |  |
| --- | --- |
| Unigene ID | Hs.99936 |

---
